# Implementation of quantum state manipulation in a dissipative cavity

**DOI:** 10.1038/srep10656

**Published:** 2015-06-22

**Authors:** Jie Song, Jing-Yan Di, Yan Xia, Xiu-Dong Sun, Yong-Yuan Jiang

**Affiliations:** 1 Department of Physics, Harbin Institute of Technology, Harbin 150001, China; 2 Department of Physics, Fuzhou University, Fuzhou 350002, China

## Abstract

We discuss a method to perform dissipation-assisted quantum state manipulation in a cavity. We show that atomic spontaneous emission and cavity decay might be exploited to drive many atoms into many-body steady-state entanglement. Our protocol offers a dramatic improvement in fidelity when noise strength increases. Moreover, the dephasing noise is suppressed effectively by showing that high-fidelity target state can be obtained in a dissipative environment.

Controlling quantum state superposition is one of the most challenging and attractive gaols in quantum information science. In reality, quantum coherence is destroyed because a quantum system couples to its destructive environment inevitably. Thus, much recent interest has focused on the manipulation of quantum state in a noisy environment^1^,^2^,^3^,^4^. With the recent progress, the idea that noise can assist the preparation of entanglement has been put forward. For example, the authors have investigated the dissipative dynamics of two-qubit system^5^,^6^,^7^. It has been shown that some initial states can be driven to steady states which have a nonzero entanglement in noisy environment^8^,^9^,^10^. Although the amount of entanglement induced by noise is small, these methods might open up a new perspective for engineering quantum state. In addition, many interesting methods have been developed to protect entanglement against decoherence^11^,^12^,^13^,^14^.

The approaches for dissipative preparation comply with the requirement of a nearly perfect fidelity of quantum state and the robustness against various types of noise. Thus a lot of novel schemes are proposed to generate a maximally entangled state of two atoms^15^,^16^ or a large W state^17^ by using dissipation in an optical cavity. Multi-qubit cluster state of the atoms is generated as a steady state through the atomic spontaneous emission^18^,^19^. On the other hand, neutral atoms provide a useful tool for investigating dissipative preparation of entanglement. Two atoms might be driven to entangled state with a high fidelity via the mechanism of Rydberg blockade^20^,^21^. In experiment, by combining unitary operations with engineered dissipation, a Bell state of two trapped ions may be produced with a fidelity of 0.892^22^. It is also necessary to devise methods to improve the fidelity of entanglement when the noise strength increases. Moreover, the fluctuation of environment might introduce dephasing noise. The noise channel will compete with the wanted dissipative channels. As a result, the dephasing noise will lead to the breakdown of the dynamics process of quantum state manipulation.

In this paper, we investigate quantum state manipulation in a cavity via engineering dissipative processes. By suitably choosing the intensities and detunings of fields, we built the effective decay channels through which the system is driven to the desired steady state. It is well known that the fidelity will be reduced with the increasing of noise strength. Using our method, high-fidelity quantum state can be obtained even when the strength of noise increases. Moreover, we are particularly interested in reducing the negative effect of dephasing noise.

We consider that many identical three-level atoms interact with a cavity. Each atom has one excited state 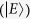 and two stable ground states (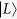 and 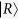). The transitions 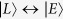 and 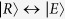 are driven by a classical field and a quantized cavity field, respectively. The parameter 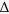 is the detuning of driving field frequency from atomic transition frequency. Another cavity mode drives the transition 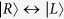 resonantly (The type of coupling mechanism has been used in^22^ and may be realized by a Raman transition via a fourth level). The Hamiltonian for the whole system is written as


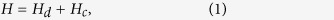


where


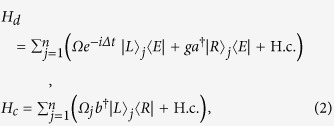


where subscript 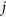 corresponds to the 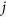
th identical atom. 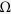 is the Rabi frequency of classical pulse. 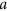 and 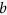 are the annihilation operators for cavity modes. 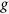 and 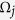 are the atom-cavity coupling constants. In the system, the source of decoherence originates from cavity photon decay and atomic spontaneous emission. With considering the dissipation within Markovian approximation, the time evolution operator is given by a master equation with a Lindblad form
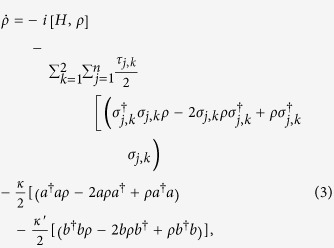
where 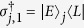 and 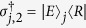. 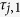 and 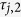 are the spontaneous emission rates which are related to the decay channels 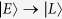 and 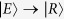, respectively. 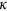 and 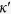 are the photon decay rates (for the sake of simplicity, we set that 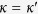). The central idea of our work can be understood by considering three atoms in an open cavity. When the ground state 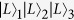 is initially populated and the strength of classical field 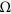 is sufficiently weak, there is only one single excitation in the whole system. The transition from 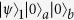 to 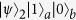 occurs with the coupling constant 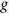. Here 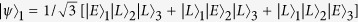 and 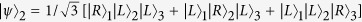. The subscripts 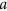 and 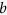 denote the cavity modes 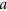 and 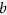, respectively. Then the excited manifold may show energy splitting. Under the condition that 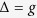, the state 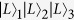 is resonantly coupled to the state 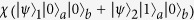 (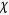 is a normalization parameter). The atoms decay through the cavity mode 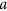 from the ground state to the state 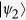 which is our wanted state. With considering the homogeneous collective spontaneous emission of atoms, the net transfer is only possible from the ground state to state 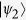. A similar behavior has been reported for the system of an optical cavity containing many atoms in the presence of collective spontaneous emission^17^ . When the other atomic decay channels are included^18^, the final state will be a mixed state instead of 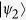. Thus the fidelity of state 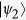 is decreased greatly. In order to obtain our wanted state, the coupling constants 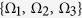 are chosen as 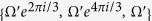 and 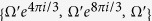 sequentially. If the atoms are not in state 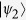, they will be transferred to the ground state. The classical field drives the atoms from ground state to excited state again. Coherent driving corresponding to 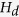 and 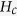 are performed repeatedly. Consequently, in the presence of symmetry breaking, the competition between the coherent and dissipative dynamics can drive the system to the steady state 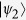. Similarly, for many-atom case, the coupling constants 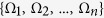 are set to be 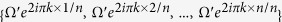 sequentially. Here 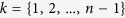. We can use this method to drive many atoms into the state with single excitation.

In Fig. 1(a), we consider the case that three atoms in a cavity are initially in ground state. By a direct numerical simulation of master equation with the Hamiltonian in Eq. (1), the temporal evolution of density matrix is calculated numerically. As expected, the numerical results show that the fidelity of state 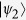 can reach 0.92. In addition, the fidelity of four-qubit state is about 0.9 if 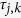 is 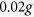 and the other parameters are chosen the same as those in three-qubit case. Without loss of generality, in the following, we will use three atoms as an example. With the increasing of 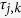 and 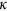, the Lindblad operators corresponding to noise terms will drive the transition from 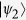 to the other states, which makes the stationary state be away from 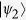, so the fidelity is reduced greatly. Similar problem arises in Refs.[15, 16, 17, 18]. Is there any way to improve the fidelity under strong dissipation?In Fig. 1(b), one can observe that the density matrix can be expressed as 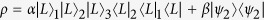 approximately because the other matrix elements have negligible amplitudes. The stronger the noise strengths are, the larger the parameter 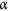 is. To improve the fidelity, the single qubit operations 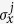 will be performed on the atom 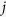 
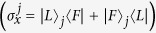
. Here 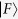 is an auxiliary ground state which is not coupled to the cavity mode. Then we set the parameters 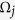 to be equal to 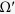. Through the driving 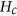, the atoms in state 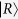 will evolve to state 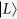. The photon in mode 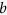 need be detected by a detector. The process is described by the following master equation


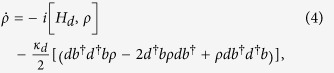


where 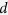 denotes the annihilation operator of a detector mode. The master equation describes an irreversible detection process. By detecting the photon at time 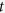 (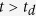), the system is projected to the subspace where the detector clicks. Correspondingly, the density matrix is expressed by


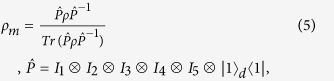


where 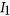, 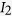, and 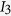 are the identity operations of atoms. 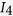 and 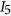 are the identity operations of cavity modes 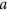 and 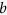, respectively. The state 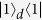 corresponds to the Fock state of detector mode. Then the target state is 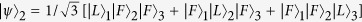. In Fig. 2, the fidelity of three-qubit is improved from 0.8 to 0.98 when the cooperativity 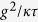 is about 20.

It is very important to discuss the variation of interaction time to reach the stationary state when the number of atoms increases. For many-atom system, we cannot simulate the dynamics evolution directly because of the increased complexity. In order to get additional insight, we use Monte Carlo wave function method^23^,^24^ to calculate the time evolution of four- and five-atom states. The simulations are performed under 200 quantum trajectories for each time point. Because the time needed to complete the preparation process is longer than its corresponding detection time, we will consider the time evolution in the preparation process. In Fig. 3(a), one observes that the fidelity of four- or five-atom state is about 0.9. The equilibration time corresponding to four- and five-atom systems is slightly different. However, with increasing of atomic number, the interaction time should increase. In Figs. 3(b,c), the density matrix elements are shown. The steady-state density matrix of the atoms can still be written as 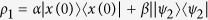 approximately. Here 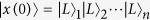, 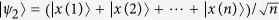 and 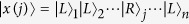 (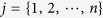). As the interaction time decreases, the ratio 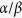 becomes large. Then high-fidelity state can still be obtained in the detection process. For example, when the interaction time is chosen as 3000/g and the other parameters are the same as those in Fig. 3, the final fidelities of four- and five-atom states are 0.99 and 0.97, respectively.

It is necessary to consider the influence of inhomogeneous dephasing on the creation of quantum state. The super-operator for dephasing noise is given as follows


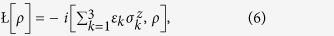


where 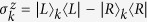 and 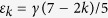. The fidelity is calculated by solving the master equation including the dephasing noise term. Unfortunately, the fidelity is significantly decreased when the dephasing noise is taken into account, i.e., the fidelity is only 0.1 (0.8) before (after) detecting the photon, if we chose 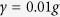 in Fig. 5(a). How do we reduce the undesired effect of dephasing noise in the whole process?Base on our method, a simple modification can be made to resist the influence of inhomogeneous dephasing noise. In the beginning, the state of atoms is driven by 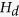 within a time interval 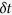. Then the 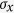 operations drive the atomic transition between the states 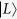 and 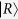. In the following, we apply 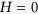 for the same time interval 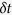. Another 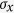 operation is performed on each atom again. That is to say, after each coherent driving over a time interval 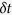, the operations 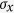, 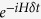, and 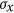 are done on the system sequentially. The basic unit of the whole process can be described by open system dynamical maps shown in Fig. (4). From Fig. 5(b), we show that the final fidelity is about 0.97 in the presence of dephasing noise. The physical mechanism can be understood as follows: the dephasing noise will induce an unwanted phase fluctuation that destroys the coherence of our target state. The unwanted phase can be cancelled by applying the operations 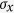, 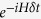, and 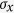 in the noisy environment. In addition, the dephasing noise results in a larger or smaller energy shift which does not change the dynamics evolution dramatically within a short time interval. We also must point out that, with the increasing of time interval, the influence of phase noise on the dynamics will no longer be neglected. However, the fidelity is more than 0.9 if the time interval is less than 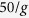.

Next we will discuss the influence of atomic spontaneous emission and photon decay on the manipulation of quantum state. Because the noise is not easy to control, the strength of noise parameter may vary within a wide range. In our work, the dissipation term for the channel 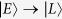 plays a neglectable role due to the fact that the atoms are driven to ground state along the channel. On the other hand, the varied spontaneous emission rate for the channel 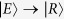 incoherently changes the distribution of populations. Thus we will fix the parameter 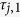 and consider the effect of the variation of spontaneous emission rate 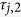 corresponding to the channel 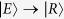. In Fig. 6(a), one finds that the final fidelity is about 0.974 when the noise parameters vary in a wide range. In Fig. 6(b), when photon decay rate 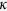 is changed from 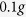 to 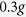, we observe that the fidelity is 0.975. The results show that our method can be robust against the variation of noise parameters.

Now we would like to give a brief analysis of the experimental implementation. The configuration of atom may be realized with existing atom-cavity system in experiment^25^,^26^. In our proposal, we chose the parameters as 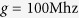, 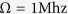, 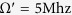, 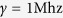, 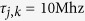, 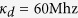 and 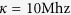, then the target quantum state is obtained with a fidelity of 0.98. The total interaction time is about 73 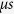, which is smaller than the lifetime of metastable state. In the detection process, the conventional detector is only required to distinguish the vacuum and non-vacuum Fock number states because the total excitation number is less than or equal to 1 under the condition of weak driving. In addition, does the imperfect efficiency of detector influence the implementation? To evaluate the effect of detection efficiency on the fidelity, the dissipative term is arranged into the Lindblad form as 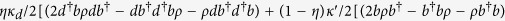. If the efficiency is low, the photon might leak into the environment. Then the detector will not be clicked. As a result, the success probability will be decreased. However, the fidelity is almost not affected, i.e., when the efficiency of detector 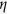 is 0.8, 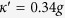, and the other parameters are the same as in Fig. 2, the fidelity and success probability for three-qubit state are about 0.98 and 0.81, respectively. Therefore, the method might be used to obtain a high-fidelity quantum state in an open system.

In conclusion, we have studied the dissipative dynamics of many atoms in a cavity. We showed that the target quantum state can be obtained by engineering the source of noise. Both cavity decay and atomic spontaneous emission have been changed from a detrimental source to a useful resource. The fidelity inevitably drops with the increasing of the noise strengths. However, the fidelity of steady state can be further improved by detecting the photon in cavity. Furthermore, a slight modification of our method allows the creation of target quantum state in the presence of dephasing noise. Thus our protocol might open up a promising perspective for manipulating quantum state in a noisy environment.

## Additional Information

**How to cite this article**: Song, J. *et al.* Implementation of quantum state manipulation in a dissipative cavity. *Sci. Rep.* **5**, 10656; doi: 10.1038/srep10656 (2015).

## Figures and Tables

**Figure 1:**
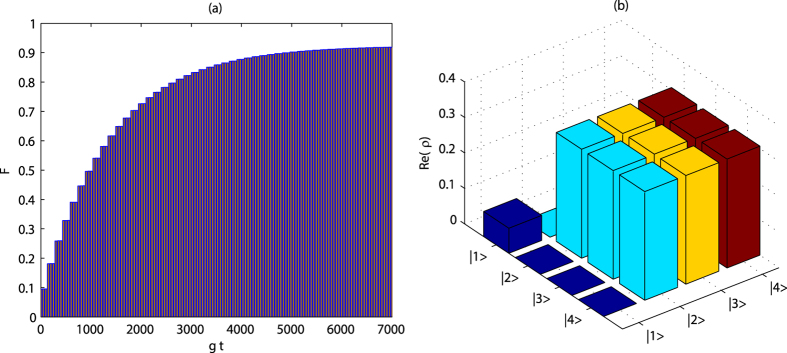
(**a**) Variation of the fidelity as a function of time. (**b**) The steady-state density matrix in the space spanned by the state vectors 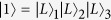, 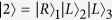, 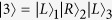, and 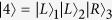. The other parameters are 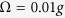, 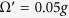, 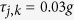, 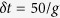, and 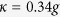. Here 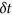 is the time period for each driving.

**Figure 2:**
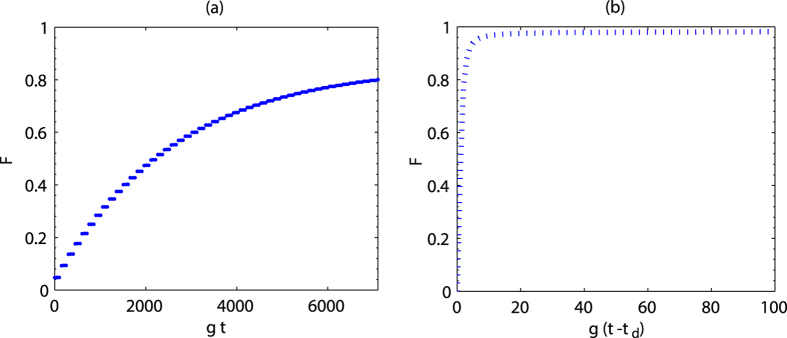
Evolution of fidelity with (**a**) interaction time and (**b**) detection time. We have taken 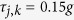, 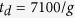, and 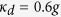. The other common parameters are the same as those in Fig. 1.

**Figure 3:**
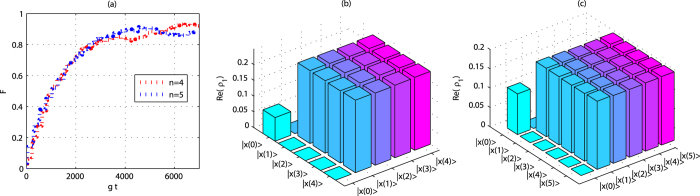
(**a**) Time evolution of fidelity versus 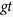 with different atom numbers 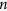. The real parts of density matrix elements of (**b**) four-atom state and (**c**) five-atom state. The spontaneous emission rates are 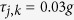 and the other parameters chosen are the same as in Fig. 2.

**Figure 4:**
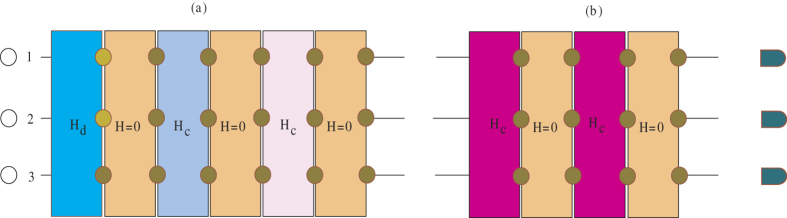
Quantum circuit for the realization of dissipative maps. The processes of implementing dissipative maps are consisted of (**a**) the elementary preparation process and (**b**) the detection process. The atoms are represented as black circles and the operations as rectangles. The yellow dots denote the operations 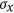.

**Figure 5:**
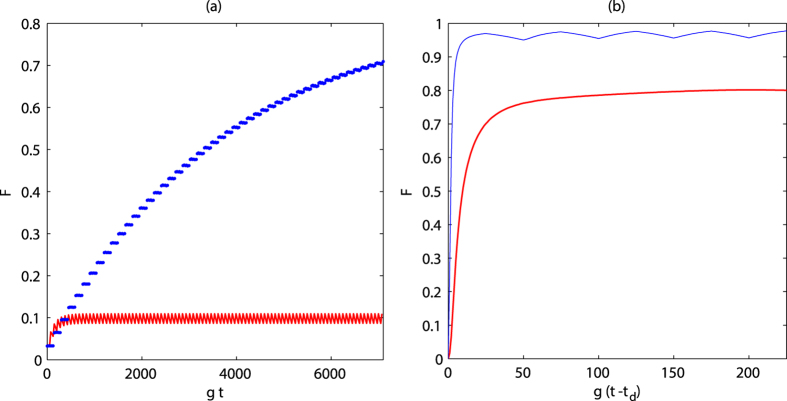
Time evolution of fidelity for 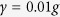 in (**a**) the preparation process and (**b**) detection process. The red/blue line corresponds to the original/modified method. Parameters set: 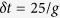 and 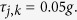 The other parameters are the same as those in Fig. 2.

**Figure 6:**
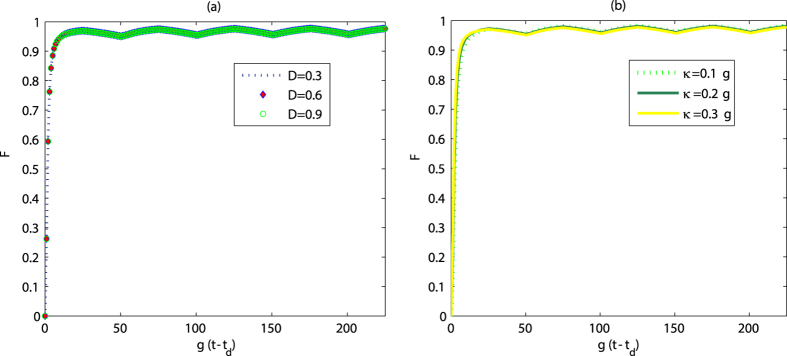
Fidelity versus time for various values of decay rate. The parameters are (**a**) 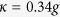; (**b**) 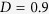. We have taken 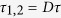, 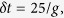 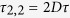, 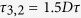, and 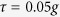. The other parameters are the same as those in Fig. 2.
